# HASwinNet: A Swin Transformer-Based Denoising Framework with Hybrid Attention for mmWave MIMO Systems

**DOI:** 10.3390/e28010124

**Published:** 2026-01-20

**Authors:** Xi Han, Houya Tu, Jiaxi Ying, Junqiao Chen, Zhiqiang Xing

**Affiliations:** 1 School of Artificial Intelligence and Computer, North China University of Technology, Beijing 100144, China; totuthy5177@gmail.com (H.T.);; 2 Department of Mathematics, Hong Kong University of Science and Technology, Hong Kong 999077, China; jx.ying@connect.ust.hk

**Keywords:** mmWave backhaul, MIMO, channel denoising, hybrid attention, Swin Transformer, deep learning

## Abstract

Millimeter-wave (mmWave) massive multiple-input, multiple-output (MIMO) systems are a cornerstone technology for integrated sensing and communication (ISAC) in sixth-generation (6G) mobile networks. These systems provide high-capacity backhaul while simultaneously enabling high-resolution environmental sensing. However, accurate channel estimation remains highly challenging due to intrinsic noise sensitivity and clustered sparse multipath structures. These challenges are particularly severe under limited pilot resources and low signal-to-noise ratio (SNR) conditions. To address these difficulties, this paper proposes HASwinNet, a deep learning (DL) framework designed for mmWave channel denoising. The framework integrates a hierarchical Swin Transformer encoder for structured representation learning. It further incorporates two complementary branches. The first branch performs sparse token extraction guided by angular-domain significance. The second branch focuses on angular-domain refinement by applying discrete Fourier transform (DFT), squeeze-and-excitation (SE), and inverse DFT (IDFT) operations. This generates a mask that highlights angularly coherent features. A decoder combines the outputs of both branches with a residual projection from the input to yield refined channel estimates. Additionally, we introduce an angular-domain perceptual loss during training. This enforces spectral consistency and preserves clustered multipath structures. Simulation results based on the Saleh–Valenzuela (S–V) channel model demonstrate that HASwinNet achieves significant improvements in normalized mean squared error (NMSE) and bit error rate (BER). It consistently outperforms convolutional neural network (CNN), long short-term memory (LSTM), and U-Net baselines. Furthermore, experiments with reduced pilot symbols confirm that HASwinNet effectively exploits angular sparsity. The model retains a consistent advantage over baselines even under pilot-limited conditions. These findings validate the scalability of HASwinNet for practical 6G mmWave backhaul applications. They also highlight its potential in ISAC scenarios where accurate channel recovery supports both communication and sensing.

## 1. Introduction

With the explosive growth of mobile data traffic and the increasing demand for high-capacity backhaul, millimeter-wave (mmWave) massive multiple-input, multiple-output (MIMO) has emerged as a key technology for next-generation networks. By exploiting the abundant bandwidth and spatial multiplexing gains, mmWave MIMO enables ultra-high data rates [1,2]. Nevertheless, its inherent path loss, sparse multipath structure, and low-rank channel characteristics pose significant challenges for accurate channel estimation and denoising. Consequently, reliable acquisition of channel state information (CSI) under constrained training overhead remains a major problem. While conventional channel estimation focuses on extracting CSI from pilot symbols using statistical methods, these methods often suffer severe performance degradation under low signal-to-noise ratio (SNR) and limited pilot overhead. Channel denoising, on the other hand, treats the initial noisy estimate as a corrupted image and aims to recover the underlying clean structure. This paper specifically addresses the practical problem of channel denoising in mmWave massive MIMO systems, where the goal is to recover accurate CSI from severely corrupted observations resulting from stringent pilot constraints and harsh propagation environments.

The introduction of reconfigurable intelligent surfaces (RIS) further enhances beamforming capabilities and coverage. Nevertheless, the passive nature of RIS makes direct estimation of individual channel components infeasible, thereby increasing the complexity of CSI acquisition [3,4]. Beyond communications, mmWave MIMO is also regarded as a promising enabler for integrated sensing and communication (ISAC), since its large antenna arrays and high-frequency operation naturally support high-resolution radar-like sensing while delivering high-capacity links. For instance, Wang et al. [5] proposed an assistant vehicle localization scheme based on three collaborative base stations via SBL-based robust DOA estimation, demonstrating the potential of high-precision sensing. Gao et al. [6] demonstrated that compressed sampling-based frameworks can jointly recover CSI and radar perception information, thereby reducing pilot overhead while enabling ISAC functionalities. This underscores that efficient channel estimation and denoising not only benefit communications but also play a key role in enabling joint sensing capabilities for sixth-generation (6G) systems.

In response to these challenges, deep learning (DL) techniques have gained considerable attention in wireless communication research. Early DL-based methods approached the physical layer holistically, employing end-to-end training to jointly estimate the channel, detect signals, and decode information [7]. Subsequent developments combined traditional signal processing with neural network-based refinement, while recurrent models like long short-term memory (LSTM) were leveraged to capture temporal channel dynamics in time-varying environments [8]. A promising strategy in this field is to treat channel estimation as a signal restoration problem, where the channel matrix is analogous to a noisy image. DL methods are then used to reconstruct a cleaner, more accurate version of the channel. Early approaches in this direction included convolutional neural network (CNN)-based denoisers and super-resolution networks [9,10], while more recent models have explored diffusion-based generative methods for structure-preserving channel recovery, offering improvements over traditional generative adversarial network (GAN)-based approaches [11,12].

Recent DL models for mmWave and RIS-assisted systems have demonstrated strong capabilities in capturing angular-domain sparsity and spatial correlations. Both compressive sensing-inspired and data-driven architectures have been explored [13]. Notable advancements include domain-specific attention mechanisms such as Channelformer [14] and Comm-Transformer [15], which integrate hierarchical attention and positional encoding to better represent channel characteristics. In RIS-assisted systems, methods like RIS-TX [16] enhance estimation by actively exciting pilot signals to overcome passive reflection challenges. Furthermore, the combination of DL and RIS has been comprehensively reviewed, and the results confirm that data-driven solutions are particularly effective for RIS-assisted wireless networks [17].

From an architectural perspective, our work builds upon the Swin Transformer [18], which introduces hierarchical shifted-window attention to balance local feature extraction and global context modeling. This design is particularly advantageous for mmWave channels, where both clustering and angular sparsity are simultaneously present. A recent tutorial article [19] has systematically reviewed Transformer-based architectures in wireless communications and emphasized their capability to model long-range dependencies. In addition, sparse representation plays an important role in mmWave channel estimation. Wang et al. [20] investigated polarization channel estimation for circular and non-circular signals in massive MIMO systems, highlighting the value of exploiting signal structures. Similarly, Zhang et al. [21] demonstrated that combining compressive sensing priors with deep neural networks significantly improves sparse channel recovery in massive MIMO systems. Motivated by this, we design a sparse token extraction module guided by angular-domain significance.

Another critical dimension is frequency-domain consistency. In practice, residual distortions often manifest as spectral mismatch, degrading performance across subcarriers. Mashhadi and Gündüz [22] developed a frequency-aware deep learning approach for joint pilot design and channel estimation in MIMO-OFDM, leveraging inter-frequency correlations to enhance performance. Inspired by this idea, we incorporate an angular-domain perceptual loss to preserve the clustered multipath structure and enhance generalization.

Despite these advances, many existing models fall short of capturing both long-range angular dependencies and local spatial structures, especially in low-SNR environments or when pilot symbols are limited. Specifically, while CNN-based methods excel at modeling localized features, they struggle with representing global context due to their limited receptive fields. Similarly, recurrent models like LSTM, although effective for temporal sequences, typically treat the 2D channel matrix as 1D sequences, thereby disrupting the intrinsic 2D spatial-angular correlations required for accurate reconstruction. Although U-Net architectures improve multi-scale feature extraction through encoder-decoder structures, they generally treat channel denoising as a generic image restoration task, often failing to exploit the specific, sparse, clustered structure of mmWave propagation. Furthermore, standard Transformer architectures, while capable of modeling global relationships, often incur quadratic computational complexity, making them prohibitive for the large channel dimensions typical of 6G backhaul systems. Moreover, few prior models explicitly decouple coarse angular patterns from dense spatial textures in the channel matrix.

To address these limitations, we propose HASwinNet, a DL framework specifically designed for mmWave MIMO channel denoising. Intuitively, the proposed system operates in three stages: First, it utilizes a hierarchical Swin Transformer to extract multi-scale spatial features from the noisy input. Second, it splits the processing into two parallel branches: a Sparse Token branch that identifies and preserves the dominant multipath components, and an Angular Refinement branch that enforces spectral consistency to filter out incoherent noise. Finally, these complementary features are fused to reconstruct a high-fidelity channel matrix. This design ensures that both the strong directional paths and the subtle angular structures are preserved. The framework is intended for deployment at the Small Cell Base Station (SCBS) receiver side to enable real-time denoising of uplink or backhaul signals.

The main contributions of this work are summarized as follows:

- We explicitly formulate the channel denoising problem distinct from traditional estimation, and propose HASwinNet, a hierarchical framework based on Swin Transformer. Unlike conventional CNNs with fixed receptive fields, our architecture efficiently models both local spatial textures and long-range angular dependencies, making it effective for capturing the clustered multipath structure of mmWave channels.
- We design a novel dual-branch mechanism that decouples signal recovery into two complementary tasks: a sparsity-aware token extraction module that identifies dominant angular components, and a spectral consistency branch that utilizes DFT/IDFT operations to filter incoherent noise. This hybrid design ensures that faithful channel reconstruction is achieved by simultaneously exploiting angular sparsity and frequency-domain coherence.
- We demonstrate through extensive simulations on Saleh–Valenzuela (S–V) channels that HASwinNet significantly outperforms representative baselines in terms of NMSE and BER. Specifically, the proposed framework exhibits superior robustness in harsh deployment scenarios characterized by low SNR and severe pilot constraints, confirming its engineering value for future 6G backhaul and ISAC systems.

## 2. System Model

**Figure 1:**
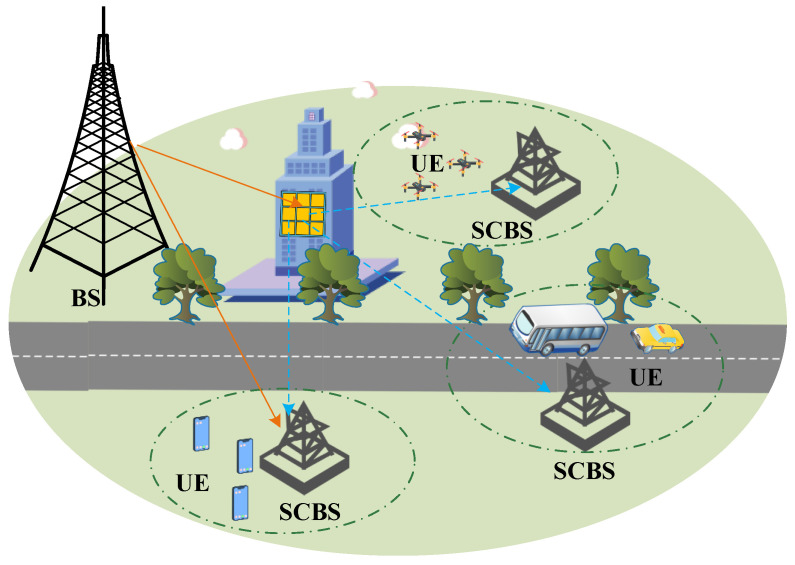
RIS-assisted mmWave MIMO backhaul system, where the BS communicates with multiple SCBSs via a passive RIS. Each SCBS serves a set of user equipments (UEs) within its local cell.

As illustrated in Figure 1, this paper considers a typical mmWave backhaul communication system comprising a macro base station (BS), a RIS, and multiple small cell base stations (SCBSs). Unlike conventional backhaul approaches that rely on optical fiber or microwave links, this architecture establishes high-speed and flexible wireless connectivity purely through mmWave communication. Due to the high sensitivity of mmWave signals to blockages, a passive RIS is introduced to enhance non-line-of-sight (NLoS) coverage, enabling indirect communication between the BS and SCBSs via intelligent reflection.

In this setup, the BS is equipped with $N_{t}$ transmit antennas, the RIS comprises *M* reconfigurable reflecting elements, and each SCBS is equipped with $N_{r}$ receive antennas. The transmission can be divided into three segments: the direct link from the BS to the RIS, the reflection and phase modulation process at the RIS, and the downstream link from the RIS to the SCBS. All propagation paths are modeled according to the S–V geometric channel model to effectively capture the sparse and directional characteristics of mmWave propagation.

Let $H_{\mathrm{BR}}\in C^{M\times N_{t}}$ denote the channel matrix between the BS and RIS, which can be expressed as follows:

(1) $$H_{\mathrm{BR}}=\sqrt{\frac{1}{P_{1}}}\sum_{p=1}^{P_{1}}\alpha_{p}^{\mathrm{BR}}\;a_{R}\left(\theta_{p}^{\mathrm{AoA}}\right)\;a_{T}^{H}\left(\phi_{p}^{\mathrm{AoD}}\right),$$

where $P_{1}$ denotes the number of propagation paths in the BS–RIS links, $\alpha_{p}^{\mathrm{BR}}$ represents the complex gain of the *p*-th path, and $a_{T}\left(\cdot \right)$ and $a_{R}\left(\cdot \right)$ are the array response vectors of the BS and RIS, respectively. The angles $\theta_{p}^{\mathrm{AoA}}$ and $\phi_{p}^{\mathrm{AoD}}$ correspond to the angle of arrival (AoA) and angle of departure (AoD) in the angular domain.

The RIS behavior is modeled via a diagonal phase-shift matrix $\Phi =\mathrm{diag}(e^{j\phi_{1}},\ldots ,e^{j\phi_{M}})$, where $\phi_{m}$ denotes the programmable phase shift at the *m*-th reflecting element. The channel between the RIS and the SCBS is denoted as $H_{\mathrm{RS}}\in C^{N_{r}\times M}$, given by the following:

(2) $$H_{\mathrm{RS}}=\sqrt{\frac{1}{P_{2}}}\sum_{p=1}^{P_{2}}\alpha_{p}^{\mathrm{RS}}\;a_{S}\left(\vartheta_{p}^{\mathrm{AoA}}\right)\;a_{R}^{H}\left(\varphi_{p}^{\mathrm{AoD}}\right),$$

where $P_{2}$ denotes the number of propagation paths in the RIS–SCBS links, and $a_{S}\left(\cdot \right)$ is the receive array response at the SCBS.

Combining the above, the end-to-end cascaded channel between the BS and SCBS, denoted as $H_{\mathrm{eq}}\in C^{N_{r}\times N_{t}}$, can be modeled as follows:

(3) $$H_{\mathrm{eq}}=H_{\mathrm{RS}}\Phi H_{\mathrm{BR}}.$$

During the pilot training phase, the BS transmits a known pilot matrix $X\in C^{N_{t}\times L}$ to probe the equivalent channel, where *L* denotes the number of pilot symbols. The received signal at the SCBS is given by the following:

(4) $$Y=H_{\mathrm{eq}}X+W,$$

where $Y\in C^{N_{r}\times L}$ denotes the received signal, and W is additive white Gaussian noise (AWGN) following the distribution $\mathrm{CN}(0,\sigma^{2}I)$. The cascaded channel $H_{\mathrm{eq}}$ explicitly depends on the RIS phase-shift matrix Φ; HASwinNet is designed to denoise the effective cascaded channel from noisy pilot observations. In our setting, Φ is not provided as an explicit side input to the network. Instead, the RIS effect is implicitly embedded in the training and evaluation data through $H_{\mathrm{eq}}$ and the received pilots Y.

To quantify the training cost, we define the dimensionless pilot overhead as

(5) $$\eta ≜\frac{L}{N_{t}},$$

which measures the proportion of pilot resources relative to the channel dimension. A larger η improves estimation accuracy but consumes more spectrum, while a smaller η preserves throughput but risks higher error. Assuming an orthogonal pilot matrix with $XX^{H}=LP_{s}I_{N_{t}}$, the least-squares (LS) estimator under (4) is as follows:

(6) $${\hat{H}}_{\mathrm{eq}}=YX^{H}{\left(XX^{H}\right)}^{-1}=H_{\mathrm{eq}}+WX^{H}{\left(XX^{H}\right)}^{-1}.$$

In this case, the additive error term has per-entry variance $\sigma^{2}/\left(LP_{s}\right)$. For the LS estimator, the NMSE is proportional to the relative estimation error power in ${\hat{H}}_{\mathrm{eq}}$, and thus scales with the same factor, yielding the following:

(7) $${\mathrm{NMSE}}_{\mathrm{LS}}\propto \frac{1}{LP_{s}}=\frac{1}{\eta \;N_{t}P_{s}}.$$

This LS-based scaling is used only for motivation and does not necessarily describe the NMSE trend of HASwinNet. This reveals a fundamental trade-off in which increasing the pilot overhead η or the pilot power $P_{s}$ reduces NMSE, while a larger overhead simultaneously decreases spectral efficiency.

Notably, mmWave MIMO and RIS-assisted channels exhibit strong angular sparsity and low-rank structures due to clustered propagation. These priors enable advanced estimators—such as compressed sensing and low-rank tensor decomposition—to recover channels reliably even when η≪1, thereby alleviating pilot overhead while maintaining low NMSE. This insight directly motivates the design of learning-based denoising frameworks that can exploit such low-rank/sparse features, as developed next in the proposed HASwinNet.

To interface with real-valued DL models, the received complex signal is separated into real and imaginary components and represented as a three-dimensional real-valued tensor:

(8) Yr=Re(Y);Im(Y)∈R2×Nr×L.

To ensure a fixed-size 2D input for the Swin-based encoder, we adopt a simple padding strategy along the pilot dimension. Specifically, when $L<N_{t}$, we zero-pad $Y_{r}$ to obtain ${\tilde{Y}}_{r}\in R^{2\times N_{r}\times N_{t}}$; when $L=N_{t}$, ${\tilde{Y}}_{r}=Y_{r}$. Unless otherwise stated, the network takes ${\tilde{Y}}_{r}$ as its input. On this basis, the proposed HASwinNet is deployed at the SCBS to reconstruct the equivalent channel from noisy observations. Formally, the network implements a data-driven denoising map:

(9) $${\hat{H}}_{r}=f_{\theta }\left({\tilde{Y}}_{r}\right),\;{\hat{H}}_{r}\in R^{2\times N_{r}\times N_{t}},$$

where $f_{\theta }\left(\cdot \right)$ denotes a learnable function parameterized by θ and ${\hat{H}}_{r}$ is the real-valued representation of the estimated channel. The complex-domain estimate is obtained by recombining the two real channels as follows:

(10) $${\hat{H}}_{\mathrm{eq}}\;=\;{\hat{H}}_{r}^{\left(1\right)}\;+\;j\;{\hat{H}}_{r}^{\left(2\right)}\;\in \;C^{N_{r}\times N_{t}}.$$

## 3. Proposed Method

The HASwinNet framework is developed to accurately recover mmWave MIMO channels under low SNR and high angular sparsity conditions. Unlike conventional CNN-based or generic Transformer-based networks, the proposed architecture incorporates Swin Transformer encoding, angular-domain refinement, and sparsity-aware attention, jointly modeling the structural properties of mmWave propagation. This design enables the network to balance global angular sparsity with fine-grained angular spectral consistency, which is essential for robust denoising in practical backhaul scenarios. The detailed forward propagation procedure of the proposed HASwinNet is summarized in Algorithm 1.
**Algorithm 1** Forward Propagation of HASwinNet.**Require:** Noisy pilot observation $Y_{r}\in R^{2\times N_{r}\times L}$, temperature τ, sparsity ratio ρ**Ensure:** Denoised channel estimate ${\hat{H}}_{r}\in R^{2\times N_{r}\times N_{t}}$**Operator definitions:** ${\mathrm{RowNorm}}_{2}{\left(V\right)}_{i}={∥V_{i,:}∥}_{2}$; TopKMask(g,K) returns a binary mask with K=⌈ρN⌉ ones. During training, we adopt a straight-through estimator (STE) for the hard mask; during inference, hard Top-*K* is used.**Stage 0: Input formatting** 1:${\tilde{Y}}_{r}\leftarrow \mathrm{Pad}\left(Y_{r}\right)$     ▹ Pad to size $2\times N_{r}\times N_{t}$ when $L<N_{t}$**Stage 1: Encoding** 2:$\chi_{0}\leftarrow \mathrm{PatchEmbed}\left({\tilde{Y}}_{r}\right)$ 3:$X_{enc}\leftarrow \mathrm{SwinEncoder}\left(\chi_{0}\right)$**Stage 2: Dual-branch processing** 4:$F_{\mathrm{ten}}\leftarrow \mathrm{DFT}\left(X_{enc}\right)$ 5:$F\leftarrow \mathrm{Reshape}(F_{\mathrm{ten}})$                  ▹$F\in C^{N\times C}$, $N=\frac{N_{r}}{P}\frac{N_{t}}{P}$*Branch A: Sparse token branch* 6:$V\leftarrow FW_{s}$ 7:$z\leftarrow {\mathrm{RowNorm}}_{2}\left(V\right)$ 8:g←Softmax(z/τ) 9:m←TopKMask(g,K)10:$V_{sel}\leftarrow \left(m⊙g\right)⊙V$11:${\hat{T}}_{\mathrm{ten}}\leftarrow \mathrm{Fold}\left(V_{sel}\right)$    ▹zero-fill + fold to full grid before IDFT12:$H_{token}\leftarrow \mathrm{IDFT}\left(\mathrm{Refine}\left({\hat{T}}_{\mathrm{ten}}\right)\right)$*Branch B: Angular refinement branch*13:$s\leftarrow \sigma (W_{2}\delta \left(W_{1}\cdot \mathrm{Pool}\left(F_{\mathrm{ten}}\right)\right))$14:${\hat{F}}_{\mathrm{ten}}\leftarrow F_{\mathrm{ten}}⊙s$15:$H_{spec}\leftarrow \mathrm{IDFT}\left({\hat{F}}_{\mathrm{ten}}\right)$**Stage 3: Fusion and reconstruction**16:$H_{fusion}\leftarrow H_{token}⊙H_{spec}$17:$H_{up}\leftarrow \mathrm{ConvTranspose}\left(H_{fusion},k=P,s=P\right)$18:$H_{skip}\leftarrow \mathrm{ResidualProj}\left({\tilde{Y}}_{r}\right)$19:${\hat{H}}_{r}\leftarrow \mathrm{OutProj}\left(H_{up}\right)+H_{skip}$20:**return **${\hat{H}}_{r}$

### 3.1. Overall Architecture

**Figure 2:**
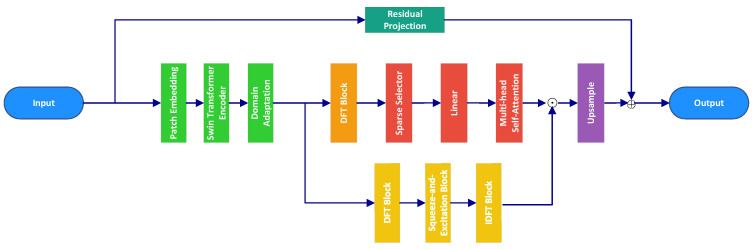
Architecture of the proposed HASwinNet denoising network.

As illustrated in Figure 2, HASwinNet adopts an encoder–decoder paradigm with auxiliary refinement paths. The input is the real-valued tensor $Y_{r}\in R^{2\times N_{r}\times L}$ formed by stacking the real and imaginary parts of the received pilot signal. A patch embedding layer first projects the raw observation into a latent feature space, followed by a Swin Transformer encoder that hierarchically extracts spatial features while preserving angular correlations across the antenna array. The encoded latent features then serve as the input for the subsequent dual-branch processing, maintaining the compressed feature representation to reduce computational redundancy.

The transformed representation is processed by two complementary branches. The first branch consists of a DFT block, a sparse selector, a linear projection, and multi-head self-attention. This pathway emphasizes dominant multipath components by projecting the angular-domain representation into a compact set of sparse tokens. The selected tokens are then refined through attention layers and upsampling, yielding a high-resolution reconstruction that captures the principal angular information. The second branch introduces spectral refinement by applying a DFT block, a squeeze-and-excitation module, and an IDFT operation. This pathway adaptively emphasizes critical frequency components and suppresses redundant noise, thereby enhancing angular-domain spectral consistency.

The outputs of both branches are fused in the decoder, while a shallow residual projection maps the original observation $Y_{r}$ directly into the channel domain, forming a stabilizing skip connection. The final channel estimate is then obtained by integrating the hybrid reconstruction with this residual pathway, as detailed in the Decoder subsection. To explicitly track the signal transformation, the tensor dimensions at each processing stage are summarized in Table 1.

### 3.2. Swin Transformer Encoder

The encoder is based on hierarchical Swin Transformer blocks. Specifically, to process the input tensor, we utilize a patch size of P=4, resulting in a grid of 16×16 tokens with an embedding dimension of C=96. The backbone comprises four stages with depths set to {2,2,6,2} and attention heads set to {3,6,12,24}, respectively. Within these blocks, we apply window-based multi-head self-attention (W-MSA) and shifted-window multi-head self-attention (SW-MSA) using a window size of M=8 and a shift size of 4. This mechanism enables both local feature extraction and long-range angular dependency modeling at a reduced computational cost compared with global attention. The encoder generates domain-adapted latent features that are structurally consistent with the MIMO channel matrix, providing an effective basis for subsequent sparse and spectral processing.

### 3.3. Sparse Token Branch

The sparse token branch explicitly exploits the angular sparsity of mmWave propagation. Given the encoded features comprising N=256 tokens on a 16×16 grid, a DFT block along the antenna dimension transforms them into the angular domain, producing $F\in C^{N\times C}$, where $N=\frac{N_{r}}{P}\frac{N_{t}}{P}=256$ and C=96. Here, the DFT is applied along the 16×16 antenna-grid dimensions $\left(\frac{N_{r}}{P},\frac{N_{t}}{P}\right)$. To filter out noise and redundancy, we enforce a sparse token budget of K=64, retaining only the top-25% most significant features. A sparsity-aware gating operator is then applied to emphasize these informative components while attenuating noise-dominated ones. Formally, this operation can be expressed as

(11) $$\hat{T}=G_{s}\;\left(F\cdot W_{s}\right),$$

where $W_{s}\in R^{C\times d}$ is a learnable projection, *d* denotes the projected token embedding dimension after the linear projection $W_{s}$. Accordingly, the sparse token set satisfies $\hat{T}\in R^{K\times d}$, and $G_{s}\left(\cdot \right)$ denotes a temperature-controlled soft gating function:

(12) $$G_{s}\left(z\right)=\frac{\exp(z/\tau )}{\sum_{j}\exp\left(z_{j}/\tau \right)},\;\tau >0.$$

Here, z represents the token importance scores, and τ is a temperature parameter. This design provides a differentiable approximation of sparse selection: with large τ, the gating weights are smooth and stable for training, while decreasing τ gradually sharpens the distribution, leading to selective emphasis on dominant angular paths. The resulting sparse tokens are subsequently refined by linear transformation and multi-head self-attention (MSA), followed by upsampling to restore spatial resolution.

### 3.4. Angular Refinement Branch

The spectral refinement branch complements the sparse pathway by enforcing angular-domain consistency. After applying a DFT block, the transformed features $F_{\mathrm{ten}}\in C^{C\times 16\times 16}$ are passed through a squeeze-and-excitation (SE) module that adaptively reweighs spectral components. Here, $F_{\mathrm{ten}}$ denotes the tensor-form angular-domain features used for spectral refinement, to avoid ambiguity with the matrix-form tokens in the sparse branch. The reweighting operation is defined as

(13) $$s=\sigma \;\left(W_{2}\;\delta \;\left(W_{1}\cdot \mathrm{Pool}\;\left(F_{\mathrm{ten}}\right)\right)\right),$$

where Pool(·) denotes global average pooling over the 16×16 angular-grid dimensions, δ(·) is the ReLU activation, σ(·) is the sigmoid activation, and $W_{1},W_{2}$ are learnable weights. The resulting $s\in R^{C}$ is broadcast along the 16×16 grid for element-wise reweighting. The refined spectral representation is then obtained as

(14) $${\hat{F}}_{\mathrm{ten}}=F_{\mathrm{ten}}⊙s,$$

which emphasizes informative angular bins and suppresses irrelevant ones. An IDFT block finally maps ${\hat{F}}_{\mathrm{ten}}$ back to the spatial domain, providing an angularly consistent refinement that complements the sparse token pathway.

### 3.5. Decoder and Reconstruction

To efficiently recover the channel structure, the decoder first performs feature fusion in the latent domain. Specifically, the token-based reconstruction ${\hat{H}}_{\mathrm{token}}$ and the spectrally refined output ${\hat{H}}_{\mathrm{spec}}$ are combined through element-wise multiplication while still at the encoded resolution of 16×16:

(15) $$H_{\mathrm{fusion}}={\hat{H}}_{\mathrm{token}}⊙{\hat{H}}_{\mathrm{spec}},$$

where ⊙ denotes the Hadamard (element-wise) product. This latent-space fusion ensures that structural consistency is enforced before expanding to the full spatial dimension.

Subsequently, to restore the full channel resolution, the fused features $H_{\mathrm{fusion}}$ are processed by a transposed convolution layer, denoted as U(·), with a kernel size and stride of 4. This operation symmetrically matches the encoder’s patch partition and directly upsamples the features back to the 64×64 spatial dimension. To stabilize training and preserve low-level information, a residual projection of the original observation $Y_{r}$ is introduced, denoted as $H_{\mathrm{skip}}$. The final channel estimate is then obtained by adding the upsampled reconstruction to the residual pathway:

(16) $${\hat{H}}_{r}=U\left(H_{\mathrm{fusion}}\right)+H_{\mathrm{skip}}.$$

This formulation integrates the recovered angular features with the raw observation, balancing the reconstruction of sparse multipath clusters with the preservation of element-wise signal details.

### 3.6. Loss Function

The proposed HASwinNet is optimized in an end-to-end manner using a composite loss that reflects the physical characteristics of mmWave propagation, namely spatial-domain fidelity, angular sparsity, and angular-domain spectral consistency. The overall loss is defined as

(17) $$L=\alpha \cdot L_{\mathrm{spatial}}\left({\hat{H}}_{\mathrm{eq}},H_{\mathrm{eq}}\right)+\beta \cdot {∥\hat{T}∥}_{1}+\gamma \cdot L_{\mathrm{ang}}\left({\hat{H}}_{\mathrm{eq}},H_{\mathrm{eq}}\right),$$

where ${\hat{H}}_{\mathrm{eq}}\in C^{N_{r}\times N_{t}}$ denotes the reconstructed channel, $H_{\mathrm{eq}}\in C^{N_{r}\times N_{t}}$ is the ground-truth channel, and $\hat{T}\in R^{K\times d}$ represents the sparse token set extracted from the encoded features after the projection $W_{s}$, where *d* is the projected token embedding dimension. The weights $\alpha ,\beta ,\gamma \in R^{+}$ are nonnegative coefficients that balance the three loss terms.

In our implementation, the weighting factors are empirically set to α=1.0, β=0.01, and γ=0.1. This specific configuration is motivated by the distinct roles of each term: α=1.0 maintains the dominance of element-wise reconstruction fidelity; a small β=0.01 provides a sufficient sparsity constraint to filter noise without eliminating weak signal components; and γ=0.1 ensures angular spectral consistency serves as a regularizer rather than a conflicting objective. We analyze the impact of varying β and γ on the denoising performance. The experimental results indicate a clear trade-off. For the sparsity weight β, setting it to 0 leads to noise residue, while increasing it beyond 0.01 degrades NMSE by over-pruning valid paths. Similarly, for the spectral weight γ, a moderate value of 0.1 yields the best structural consistency. Excessive weighting was found to destabilize training, as spectral loss began to dominate the spatial reconstruction loss.

The first term $L_{\mathrm{spatial}}$ is chosen to be the NMSE between the estimated and true channel matrices, ensuring element-wise reconstruction fidelity. The second term $∥\hat{T}{∥}_{1}$ is an $\ell_{1}$ regularization penalty that enforces sparsity in the token representation, thus selecting only the dominant angular components that correspond to strong propagation paths. The third term is an angular-domain consistency loss, expressed as

(18) $$L_{\mathrm{ang}}={∥\;|F\left({\hat{H}}_{\mathrm{eq}}\right)|-|F\left(H_{\mathrm{eq}}\right)|\;∥}_{F}^{2},$$

where F(·) denotes the two-dimensional discrete Fourier transform (DFT); |·| is the element-wise magnitude operator; and ${∥\cdot ∥}_{F}$ is the Frobenius norm. By enforcing similarity in the spectral magnitudes, this term preserves the clustered multipath structure that governs mmWave channels.

Each term in the loss plays a complementary role. The NMSE ensures accurate element-wise recovery, but may overlook structural coherence. The sparsity regularizer encourages the network to identify only the most salient paths, consistent with the limited number of dominant multipath components in physical propagation. The angular-domain term prevents angular spectral distortion, aligning the reconstructed channel with the clustered multipath structure of the ground truth. Together, these three terms strike a balance between local accuracy and global consistency, yielding superior robustness under low SNR and limited-pilot conditions.

### 3.7. Inference and Complexity Analysis

During inference, HASwinNet leverages the synergy of its sparse token branch and angular refinement branch to achieve both accuracy and efficiency. The decoder output is obtained through the element-wise fusion with residual addition, as already defined in (15) and (16). This design ensures that the fused high-level structures are preserved while the residual pathway stabilizes training and guarantees low-level fidelity.

In terms of complexity, HASwinNet benefits from both windowed attention and sparse token refinement. The window-based self-attention reduces the quadratic complexity of global attention from $O(N^{2})$ to $O(Nw^{2})$, where *N* is the input sequence length, and *w* is the window size. Further reducing the effective sequence length to K≪N yields a final complexity of $O(Kw^{2})$. To quantitatively validate this efficiency, we evaluate the model under the main experiment settings with an input size of 64×64, patch size P=4, embedding dimension C=96, window size M=8, and depths of {2,2,6,2}. In this configuration, HASwinNet contains approximately 1.52 M learnable parameters and requires 0.84 GFLOPs. The corresponding model weight size is compact, occupying about 6.1 MB (FP32) or 3.0 MB (FP16). This favorable scaling and low resource consumption highlight the practicality of HASwinNet for real-time deployment in 6G backhaul scenarios.

## 4. Performance Evaluation

Performance evaluation of the proposed HASwinNet framework is carried out in a mmWave MIMO backhaul scenario based on the S–V clustered channel model.

### 4.1. Dataset and Simulation Setup

In the simulation setup, a narrowband backhaul link is considered, with both the transmitter and receiver employing uniform linear arrays of $N_{t}=N_{r}=64$ antennas. The channel is composed of 3 spatial clusters, each generating 10 multipath components with an angular spread of 10°. The carrier frequency is set to 28 GHz, and the path loss exponent is 2.5. Link distances are uniformly distributed between 10 and 100 m.

Each sample contains a noisy observation $Y\in C^{N_{r}\times L}$ and the ground-truth channel $H_{\mathrm{eq}}\in C^{N_{r}\times N_{t}}$. For the full-pilot setting, we use $L=N_{t}=64$; for reduced-pilot experiments (e.g., half pilots), we set L=32 and pad $Y_{r}$ to ${\tilde{Y}}_{r}\in R^{2\times 64\times 64}$ before feeding it into HASwinNet. The dataset includes 50,000 training samples, 5000 for validation, and 5000 for testing. AWGN is introduced across SNR values ranging from −5 dB to 20 dB.

In data generation, each sample is synthesized by first drawing $H_{BR}$ and $H_{RS}$ according to the adopted channel model, and then constructing the effective cascaded channel $H_{\mathrm{eq}}$. The RIS phase shifts in Φ are generated following the considered hardware setting; therefore, the impact of RIS is naturally embedded in the statistics of the paired data $\left(Y_{r},H_{\mathrm{eq}}\right)$ used for training and evaluation.

### 4.2. Training Configuration

The HASwinNet model is trained using the Adam with decoupled weight decay (AdamW) optimizer with an initial learning rate of $3\times 10^{-4}$ and weight decay of $1\times 10^{-5}$. Training proceeds for 100 epochs with a batch size of 256. A warm-up phase is applied for the first five epochs, followed by a cosine annealing scheduler. The total loss function includes three components: NMSE loss, a sparsity-inducing regularization term, and an angular-domain perceptual loss based on 2D DFT magnitude. Unless otherwise stated, inference follows the same HASwinNet configuration as training.

### 4.3. Baseline Models

To evaluate the effectiveness of the proposed denoising framework, comparisons are made against three representative DL baselines widely adopted in prior literature. The first is a CNN consisting of three residual convolutional blocks tailored for local feature extraction. The second is an LSTM network that interprets the antenna columns of the channel matrix as temporal sequences, enabling sequence modeling. The third baseline is a U-Net-based model with an encoder–decoder architecture and skip connections, designed to recover spatial details at multiple scales. For fair comparison, all baseline models are constructed with comparable parameter counts and trained using the same dataset, batch size, and optimization schedule. Moreover, their hyperparameters, including learning rate, layer depth, and dropout probability, are independently optimized within a unified search space.

### 4.4. Evaluation Metrics

The denoising and channel recovery performance is assessed based on two commonly used evaluation metrics.

The normalized mean squared error (NMSE) measures reconstruction fidelity and is defined as

(19) $$\mathrm{NMSE}=E\left[\frac{∥\hat{H}{-H∥}_{F}^{2}}{{∥H∥}_{F}^{2}}\right],$$

where $\hat{H}$ and H denote the estimated and ground-truth channel matrices, respectively, E[·] denotes expectation over channel realizations, and ${∥\cdot ∥}_{F}$ denotes the Frobenius norm. This metric quantifies the average squared difference between the estimated and true channel matrices, normalized by the energy of the ground-truth channel.

The bit error rate (BER) is computed by transmitting randomly generated quadrature phase-shift keying (QPSK) symbols through the estimated channel and comparing the received symbols with the original transmitted ones. It is defined as

(20) $$\mathrm{BER}=\frac{N_{e}}{N_{\mathrm{sym}}},$$

where $N_{e}$ denotes the number of erroneously detected symbols, and $N_{\mathrm{sym}}$ is the total number of transmitted symbols.

These two metrics together reflect the recovery accuracy and communication reliability of the proposed HASwinNet model.

### 4.5. Performance Analysis

**Figure 3:**
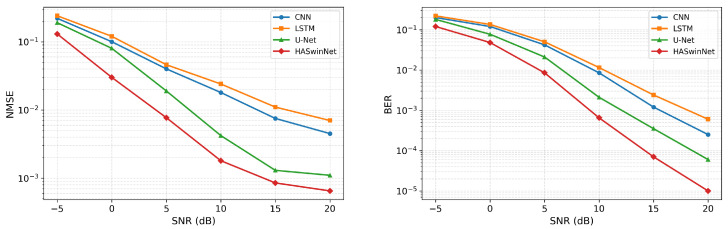
Denoising performance of different models under varying input SNRs with full pilot overhead. (**Left**): NMSE. (**Right**): BER. Each curve shows the mean of five runs.

Figure 3 presents the denoising performance of all models across input SNRs in terms of NMSE and BER. Each data point is averaged over five independent runs with different random seeds.

In the NMSE plot (left), the proposed HASwinNet consistently achieves the lowest error across the entire SNR range. Its curve decreases rapidly with increasing SNR, reaching values on the order of $10^{-3}$ at 20 dB, which is nearly an order of magnitude lower than CNN and LSTM. U-Net performs better than CNN and LSTM, but HASwinNet still maintains a clear margin, confirming its superior capability in suppressing residual noise and preserving angular-domain structures.

In the BER plot (right), HASwinNet also exhibits the best performance, with the steepest decline as SNR improves. At low SNR (−5 to 0 dB), CNN and LSTM curves are close, while U-Net achieves slightly better results. In the mid-to-high SNR regime (10–20 dB), U-Net clearly surpasses CNN and LSTM, but HASwinNet consistently achieves the lowest BER, reaching the order of $10^{-5}$ at 20 dB and thus ensuring the most reliable symbol detection.

**Figure 4:**
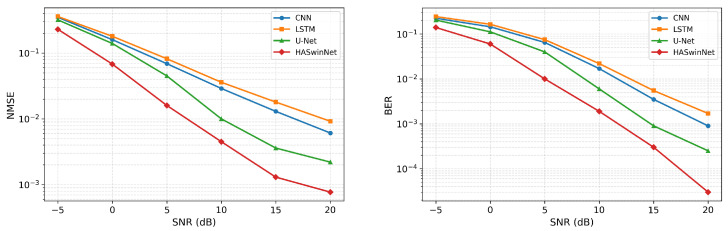
Denoising performance of different models when only half of the pilot symbols are available. (**Left**): NMSE. (**Right**): BER. Each curve shows the mean of five runs.

To further examine robustness under limited pilot resources, Figure 4 reports performance with only half of the pilot symbols. As expected, all models degrade under reduced overhead, and the relative ranking remains unchanged. HASwinNet maintains a clear margin, particularly at medium to high SNRs, with markedly smaller degradation than the baselines. This behavior aligns with the network’s hybrid design in which the sparse token pathway concentrates representation on dominant angular components, the DFT–SE–IDFT refinement preserves clustered angular spectra, the hierarchical Swin encoder captures both local detail and long-range angular dependencies, and the residual projection safeguards low-level fidelity. Together with a composite objective that enforces spatial NMSE fidelity, token-level sparsity, and angular domain spectral consistency, these elements provide strong inductive priors that allow limited pilot observations to be translated into robust reconstructions and reflect higher pilot efficiency and resilience to reduced training overhead.

**Figure 5:**
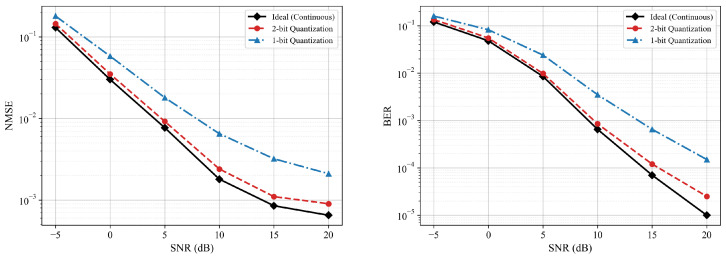
Performance comparison of HASwinNet under ideal continuous phase shifts versus practical discrete phase shifts (1-bit and 2-bit). (**Left**): NMSE. (**Right**): BER.

We further evaluate the robustness of the proposed HASwinNet against non-ideal RIS hardware constraints, specifically discrete phase shifts. Practical RIS elements often support only a limited number of phase levels due to hardware complexity and cost. Figure 5 compares the NMSE and BER performance of HASwinNet under the ideal continuous phase assumption against practical 1-bit and 2-bit quantization schemes.

As observed in Figure 5, while 1-bit quantization leads to a noticeable performance gap due to large phase errors, the 2-bit quantization scheme achieves performance highly comparable to the ideal continuous case across the entire SNR range. Notably, the performance curves of the 2-bit scheme closely follow the ideal baseline, maintaining the same order of magnitude in both NMSE and BER, even in the high SNR regime. This marginal degradation demonstrates that HASwinNet is highly robust to quantization errors and can be effectively deployed with low-cost, low-resolution RIS hardware without significantly compromising system performance.

## 5. Conclusions

This paper proposes HASwinNet, a DL-based denoising framework for mmWave MIMO channels. The architecture integrates a hierarchical Swin Transformer encoder with two complementary branches: A sparse token extraction path that captures dominant angular-domain features, and an angular-domain refinement path that enforces angular-domain consistency through adaptive reweighting. A composite loss function, consisting of spatial NMSE, angular-domain consistency, and sparsity regularization, ensures that both global and local channel characteristics are faithfully reconstructed. This design explicitly exploits the sparse multipath nature of mmWave propagation and strikes a balance between reconstruction fidelity and computational efficiency.

Extensive simulations on S–V channels have demonstrated that HASwinNet consistently outperforms representative deep learning baselines, including CNN, LSTM, and U-Net, across multiple evaluation metrics. The proposed model achieves superior NMSE and BER performance, exhibits strong robustness under low SNR conditions, and delivers pronounced gains in the high SNR regime. Moreover, experiments with reduced pilot symbols verify that HASwinNet effectively exploits angular sparsity and preserves channel structure, thereby achieving higher pilot efficiency and resilience to limited training overhead. And the proposed framework demonstrates strong robustness to hardware imperfections, maintaining high reconstruction accuracy even under low-resolution (2-bit) RIS phase quantization. These results confirm that the hybrid integration of sparse-token extraction and angular-domain refinement attains a favorable balance between reconstruction fidelity and computational complexity, underscoring the suitability of HASwinNet for practical deployment in 6G mmWave backhaul systems.

Given that mmWave massive MIMO is widely recognized as a cornerstone technology for ISAC, the proposed framework not only enhances the reliability of mmWave backhaul links but also enables accurate channel recovery that can support high-resolution sensing. Future research will extend HASwinNet to broadband and time-varying orthogonal frequency division multiplexing (OFDM) systems and conduct rigorous over-the-air (OTA) validation under realistic propagation conditions to further substantiate its applicability in unified ISAC scenarios.

## Figures and Tables

**Table 1:** Tensor dimension tracking across HASwinNet processing stages. (Settings: $N_{r}=N_{t}=64$, patch size P=4, embedding dim C=96).

Processing Block	Tensor Notation	Shape
1. Input Stage		
Pilot Observation	$Y_{r}\in R^{2\times N_{r}\times L}$	2×64×L
Padding (if $L<N_{t}$)	${\tilde{Y}}_{r}\in R^{2\times N_{r}\times N_{t}}$	2×64×64
Patch Embedding	$X_{0}\in R^{C\times \frac{N_{r}}{P}\times \frac{N_{t}}{P}}$	96×16×16
2. Encoder Backbone		
Swin Encoder Output	$X_{\mathrm{enc}}\in R^{C\times 16\times 16}$	96×16×16
3. Dual-Branch Processing		
Token Flattening	$T\in R^{N\times C}$	256×96
Sparse Selection	$\hat{T}\in R^{K\times d}$	64×96
Spectral Branch	$F_{\mathrm{ten}}\in C^{C\times 16\times 16}$	96×16×16
4. Decoder & Output		
Branch Fusion	$H_{\mathrm{fusion}}\in R^{C\times 16\times 16}$	96×16×16
Upsampling	$H_{\mathrm{up}}\in R^{C\times N_{r}\times N_{t}}$	96×64×64
Final Reconstruction	${\hat{H}}_{r}\in R^{2\times N_{r}\times N_{t}}$	2×64×64

## Data Availability

The original contributions presented in this study are included in the article. Further inquiries can be directed to the corresponding author.

## Abbreviations

The following abbreviations are used in this manuscript:

6G	Sixth-Generation Mobile Networks
mmWave	Millimeter Wave
MIMO	Multiple-Input, Multiple-Output
RIS	Reconfigurable Intelligent Surface
SCBS	Small Cell Base Station
BS	Base Station
UE	User Equipment
ISAC	Integrated Sensing and Communication
S–V	Saleh–Valenzuela
AoA	Angle of Arrival
AoD	Angle of Departure
AWGN	Additive White Gaussian Noise
LS	Least Squares
OFDM	Orthogonal Frequency Division Multiplexing
QPSK	Quadrature Phase Shift Keying
SNR	Signal-to-Noise Ratio
NMSE	Normalized Mean Squared Error
BER	Bit Error Rate
CSI	Channel State Information
DL	Deep Learning
CNN	Convolutional Neural Network
LSTM	Long Short-Term Memory
U-Net	U-shaped Convolutional Neural Network
GAN	Generative Adversarial Network
NLoS	Non-line-of-sight
MSA	Multi-Head Self-Attention
W-MSA	Window-Based Multi-Head Self-Attention
SW-MSA	Shifted Window Multi-Head Self-Attention
MLP	Multilayer Perceptron
DFT	Discrete Fourier Transform
IDFT	Inverse Discrete Fourier Transform
SE	Squeeze-and-Excitation
OTA	Over-the-Air
AdamW	Adam with Decoupled Weight Decay
